# Assessing the therapeutic potential of rutin in alleviating symptoms of inflammatory bowel disease: a meta-analysis

**DOI:** 10.3389/fphar.2025.1539469

**Published:** 2025-04-30

**Authors:** Zhenkai Zou, Fanglian Zhang, Zhigang Pan, Jinyao Xu, Xuanyi Li, Xiangjun Sun, Xufeng Wang, Feng Qin, Abulikemu Abulizi, Qian Chen, Ruicheng Yan

**Affiliations:** ^1^ The First Clinical Medical School, Hubei University of Chinese Medicine, Wuhan, China; ^2^ General Surgery Department of Honghu Hospital of Traditional Chinese Medicine, Hubei, Honghu, China; ^3^ Department of Hepatobiliary Surgery, Affiliated Hospital of Hubei University of Chinese Medicine, Hubei Provincial Hospital of Traditional Chinese Medicine, Wuhan, China; ^4^ Department of Gastrointestinal Surgery, Hubei Provincial Hospital of Traditional Chinese Medicine, Wuhan, China

**Keywords:** inflammatory bowel disease, IBD, meta-analysis, rutin, anti-inflammatory effects

## Abstract

**Introduction:**

Inflammatory bowel disease (IBD) is a chronic, potentially carcinogenic condition with limited treatment options. Rutin may represent a novel approach to treating IBD. Our aim is to conduct a systematic review of rutin, summarize its preclinical effects, demonstrate its efficacy, and provide guidance for future research.

**Methods:**

To systematically evaluate the effectiveness and feasibility of rutin in the treatment of inflammatory bowel disease (IBD) based on published experiments. A literature search up to 2023 on rutin’s effects in IBD was conducted using PubMed, Embase, and Web of Science. Three independent assessors ensured objectivity by selecting and evaluating study quality and extracting data. A meta-analysis was performed using Review Manager 5.3 and STATA 17.0, combining results for a robust evaluation of rutin’s efficacy in IBD treatment.

**Results:**

The meta-analysis includes nine animal studies with a total of 174 animals. The findings from these studies indicate that rutin has a significant positive impact on various indicators of intestinal disease caused by IBD. Key results include: reduced weight loss, lower disease activity index (DAI), decreased inflammatory markers, reduced oxidative stress markers, increased antioxidant defenses. Its mechanism of action involves anti-inflammatory, antioxidant, inhibition of inflammatory signaling pathways, barrier protection, inhibition of adaptive immune responses, restoration of intestinal permeability, and regulation of microbiota.

**Discussion:**

Preclinical evidence suggests that rutin can significantly alleviate the abnormal indicators of intestinal inflammation. In experiments, the performance of rutin in various indicators is very close to existing positive control drugs such as sulfasalazine and budesonide, with similar therapeutic effects. Taken together, this meta-analysis reveal that rutin has the potential to be a feasible drug for the treatment of IBD.

**Systematic Review Registration:**

CRD42024519891.

## 1 Background

As a chronic inflammatory disease primarily affecting the gastrointestinal tract, inflammatory bowel disease (IBD) mainly includes two types: ulcerative colitis (UC) and Crohn’s disease (CD). The exact cause of IBD is not fully understood, but it is believed to result from a combination of genetic predisposition and environmental factors, and it is most commonly diagnosed in adolescents, though it can occur at any age (Rosen et al., 2015). Among the most common symptoms experienced by individuals with IBD are persistent weight loss, rectal bleeding, fever, shortening of the colon, and the most serious risks of IBD is its potential to progress to colorectal cancer (Kim and Chang, 2014). Furthermore, the increasing global prevalence of IBD highlights the urgent need for effective treatments (Molodecky et al., 2012; Zuo et al., 2018). Common treatments include aminosalicylates, immunomodulators, corticosteroids, and anti-inflammatory cytokine antagonists (De Cassan et al., 2012; Khan et al., 2011; Nikfar et al., 2009; Rahimi et al., 2007; Muvhulawa et al., 2022).

Although these medications are effective for many patients, some may experience adverse reactions to certain drugs, which could potentially lead to the progression of other diseases (Muvhulawa et al., 2022; Carvalho et al., 2020).

Additionally, they may also cause opportunistic infections, bone marrow suppression, malignancies secondary to immunosuppression, and reactions during administration (Papamichael et al., 2020).Therefore, there is a need for new, more effective, and better-tolerated treatment measures for managing IBD.

In recent years, There is a growing interest in bioactive compounds derived from medicinal plants and food sources, which are recognized for their potent antioxidant and anti-inflammatory properties.

Rutin is a promising bioactive compounds. As a flavonol derivative, it also known as rutin glycoside, vitamin P, quercetin-3-o-rutinoside, and sophoretin. It is found in various medicinal plants and food sources and has demonstrated therapeutic effects on multiple metabolic diseases (Pandey et al., 2021).

Existing preclinical studies have provided substantial evidence supporting the beneficial effects of rutin. Its antioxidant properties are effective in reducing inflammation by lowering the levels of pro-inflammatory markers such as tumor necrosis factor-alpha (TNF-α), interleukin-6 (IL-6), and interleukin-1β (IL-1β). Rutin also inhibits the activation of nuclear factor-kappa B (NF-κB) and mitogen-activated protein kinases (MAPK), both of which play key roles in inflammatory responses. Food sources rich in rutin, such as buckwheat, arabica coffee, and loquat, have shown promise in reducing oxidative stress and inflammatory markers, highlighting the potential of dietary intake of rutin in promoting health (Muvhulawa et al., 2022). Many studies have described several pharmacological properties of rutin, including its anti-parasitic, antibacterial, anti-inflammatory, anti-tumor, antiviral, anti-allergic, vasorelaxant, cytoprotective, antispasmodic, lipid-lowering, hypotensive, and antiplatelet properties (Pandey et al., 2021). The aim of this study is to conduct a systematic review and meta-analysis of the protective effects of rutin on inflammatory bowel disease (IBD). By synthesizing existing preclinical evidence, this study aims to provide a comprehensive understanding of rutin’s potential in alleviating IBD symptoms and reducing inflammation. These findings could inform future clinical trials and contribute to the development of rutin-based therapeutic strategies for IBD.

## 2 Methods

This manuscript adheres to the reporting guidelines established by the Preferred Reporting Items for Systematic Reviews and Meta-Analyses (PRISMA) statement (Moher et al., 2009).

### 2.1 Search strategy

This study systematically searched the PubMed, Embase, and Web of Science databases for all articles published before December 2023. MeSH terms and free-text terms were combined to identify the disease and drug interventions. For example, in PubMed, such as ((“Inflammatory Bowel Diseases” [Mesh]) OR (Inflammatory Bowel Disease)) OR (Bowel Diseases, Inflammatory) AND “Rutin” [Mesh].

### 2.2 Inclusion and exclusion

This study included all existing animal experiments that assessed the effects of rutin on IBD. The selection process was conducted irrespective of the species, age, or gender of the animals used in the experiments, and met the following inclusion criteria: (1) Studies conducted in animals induced with inflammatory bowel disease in any manner; (2) Only studies in which rutin was administered as the sole treatment were included. Control groups received either a non-functional fluid, such as saline, or no treatment; (3) Irrespective of the dosage, route of administration, method, or treatment regimen of rutin; (4) Articles published within the last 10 years.

Exclusion criteria were as follows: (1) Studies categorized as opinions, reviews, case reports, or abstracts; (2) Lack of a control group or no rutin or other intervention in the treatment group; (3) Non-in vivo studies; (4) Inability to obtain full-text articles; (5) Data duplication or lack of sample size information or unavailable data; (6) Studies with incomplete experimental designs. (7) Published before 2013.

### 2.3 Data extraction

Two authors independently extracted the data to maintain objectivity and minimize errors. The extracted data included: (1) Publication year and first author; (2) IBD modeling method; (3) Animal species, gender, weight, quantity; (4) Medication dosage in the treatment group; (5) Sample size of animals; (6) Quantifiable outcome indicators, including weight loss, colon length (CL), disease activity index (DAI), interleukin-1β (IL-1β), interleukin-6 (IL-6), superoxide dismutase (SOD), malondialdehyde (MDA), tumor necrosis factor-α (TNF-α), myeloperoxidase (MPO), inducible nitric oxide synthase (mRNA iNOS), and FITC intestinal permeability. If results were only presented in figures or tables, the authors of those studies were contacted to request the specific data. If there was no response received from the authors, the WebPlotDigitizer 4.5 software (https://automeris.io/WebPlotDigitizer) was used to quantify data presented in graphical form. In studies with multiple reporting periods, data from the final time point were selected to ensure consistency in the meta-analysis. In studies where the treatment group was administered multiple doses of rutin, each dose was treated as an independent experiment.

### 2.4 Quality assessment

The SYCLE (Systematic Review Centre for Laboratory animal Experimentation) tool was selected as the framework for assessing the risk of bias in the included animal studies (Hooijmans et al., 2014), two independent assessors were tasked with evaluating each included study. Each study was scored on a scale of up to 10 points. In instances where there were discrepancies or disagreements between the assessors during the quality assessment process, the corresponding author will be consulted.

### 2.5 Statistical analysis

Statistical analysis was conducted using ReviewManager 5.3 and STATA 17.0. Each outcome measure assessed in the included studies was treated as a continuous variable for the purposes of statistical analysis, and the cumulative effect size was expressed as standardized mean differences (SMD) along with 95% confidence intervals (CI). When the original data were not in the SMD format, they were converted using ReviewManager 5.3 software. Statistical heterogeneity was assessed using I^2^, and all data were combined using a random-effects model. To explore potential sources of heterogeneity and ensure the robustness of the primary outcomes, subgroup analyses were conducted. To assess the stability and reliability of the overall results, we conducted a sensitivity analysis. Additionally, to evaluate potential publication bias in the outcome measures, we performed an Egger’s test. In cases of publication bias, the trim-and-fill method was used for correction.

## 3 Result

### 3.1 Study inclusion

**FIGURE 1:**
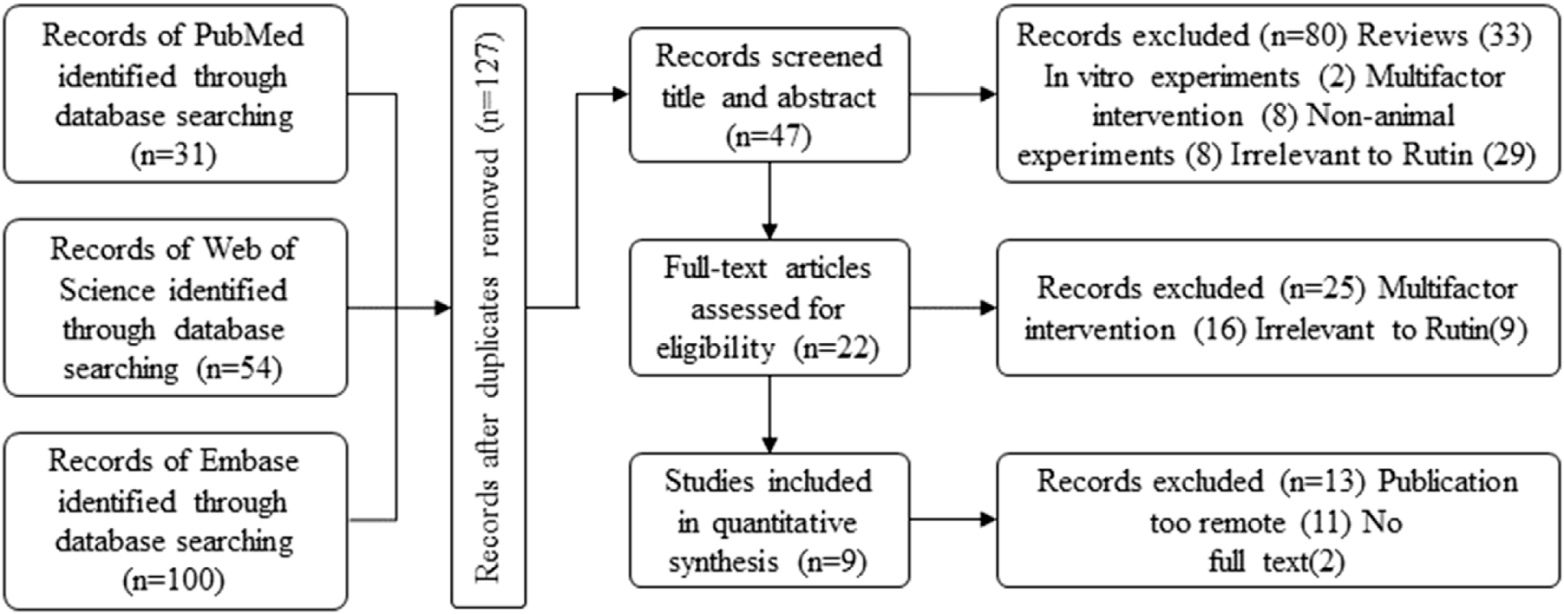
Flowchart of literature collection.

In the initial search process, 185 potentially relevant articles were identified across the databases:PubMed:31, Web of Science:54, Embace:100 (Figure 1 Flowchart of Literature Collection). After compiling the initial search results, duplicates were identified and removed to streamline the dataset, 127 articles were initially included. Among the remaining articles, 80 records were excluded based on title and abstract review. Further screening after full-text reading resulted in the exclusion of articles published more than 10 years ago. Finally, 9 studies met our inclusion criteria, all of which were published between 2013 and 2023 (within the past decade). (Marín et al., 2013; Mascaraque et al., 2014; Mascaraque et al., 2015; Abdel Ghaffar et al., 2016; Power et al., 2016; Sharma et al., 2021; Liu et al., 2022; Wang et al., 2022; Wu et al., 2023).

### 3.2 Characteristics of included studies

The 9 included studies were published in English. They involved 174 IBD model animals, including 78 in the IBD control group and 96 in the rutin treatment group. Two studies utilized Wistar rats. Four studies used C57BL/6 mice. Two studies utilized C57BL/6J mice. Two studies utilized Balb/c mice. One study utilized Rag1^−/−^ mice. Regarding the sex of the experimental animals, three studies used males and six studies used females.

Seven studies mentioned the weight of the animals, with rats weighing between 175 and 225 g and mice weighing predominantly between 18 and 20 g. Regarding the construction of IBD models, one study used the Trinitrobenzene Sulfonic Acid (TNBS)-induced model, one study used the Transfer Colitis Model (CD4^+^ CD62L + T cell transfer-induced), and the rest of the study used the DSS-induced model. The administration duration was concentrated at 7 days, with rutin doses ranging from 25 to 40 mg/kg. The outcome indicators were used to evaluate rutin’s effects on inflammatory bowel disease including body weight (7 articles), colon length (4), disease activity index (DAI) (3 articles), IL-6 (2 articles), IL-10 (2 articles), IL-1β (2 articles), TNF-α in (7 articles), MPO activity (4 articles), MDA (2 articles), mRNA iNOS (3 articles), GSH (4 articles), SOD (3 articles), and CAT (3 articles).

### 3.3 Quality of included studies

**FIGURE 2:**
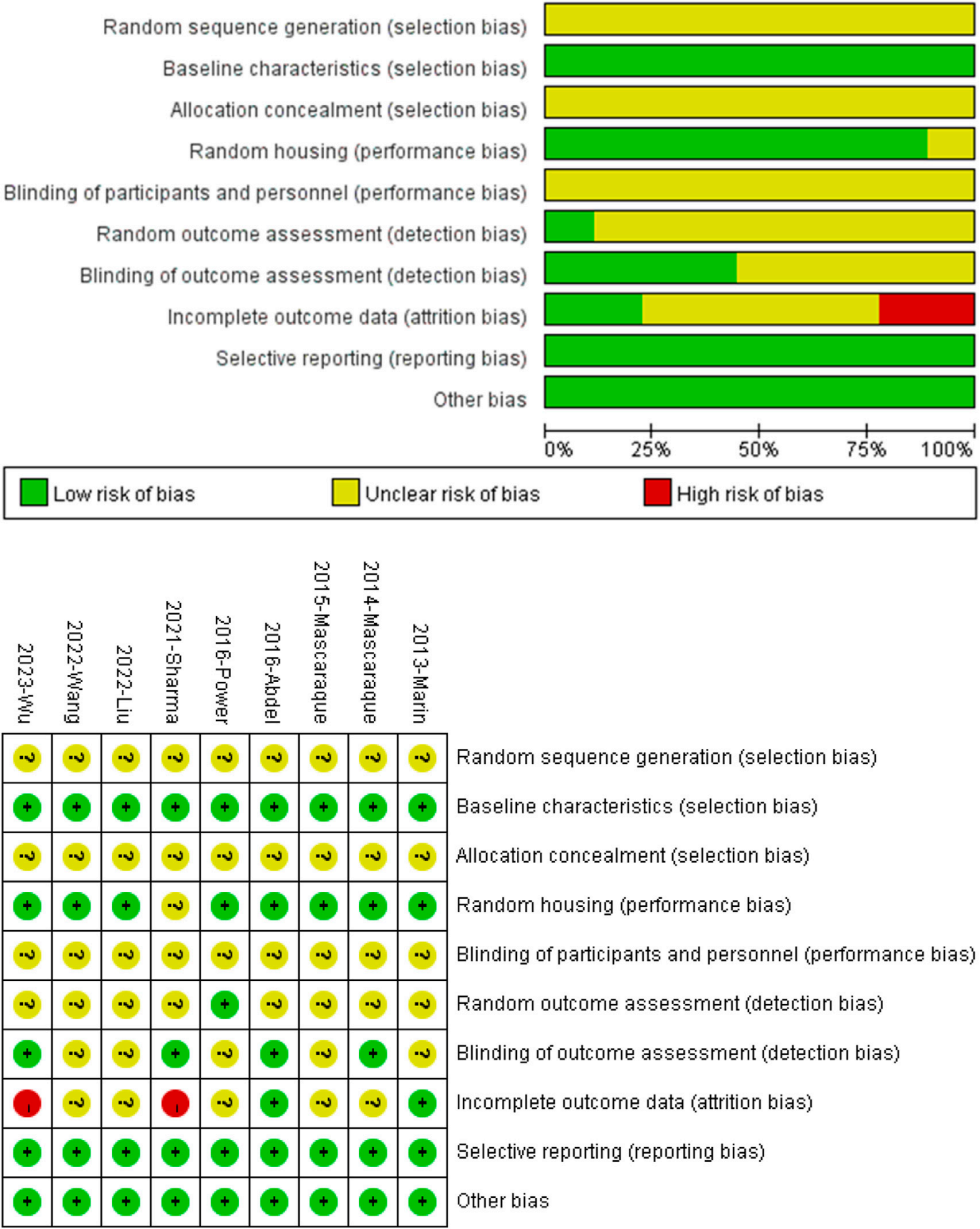
Quality of included studies.

All nine studies mentioned random grouping of animals. However, none of the studies provided information on the specific methods used to generate the random sequences, and none of the studies mentioned the specific methods of blinding for experimental grouping or the assessors. One study mentioned random outcome assessment, and four studies mentioned blinding in outcome assessment. Two studies’ results were incomplete. The authors of this paper did not identify selective reporting or other apparent biases (Figure 2 Quality of included studies.

### 3.4 The effects of rutin on IBD

#### 3.4.1 Weight loss

**FIGURE 3:**
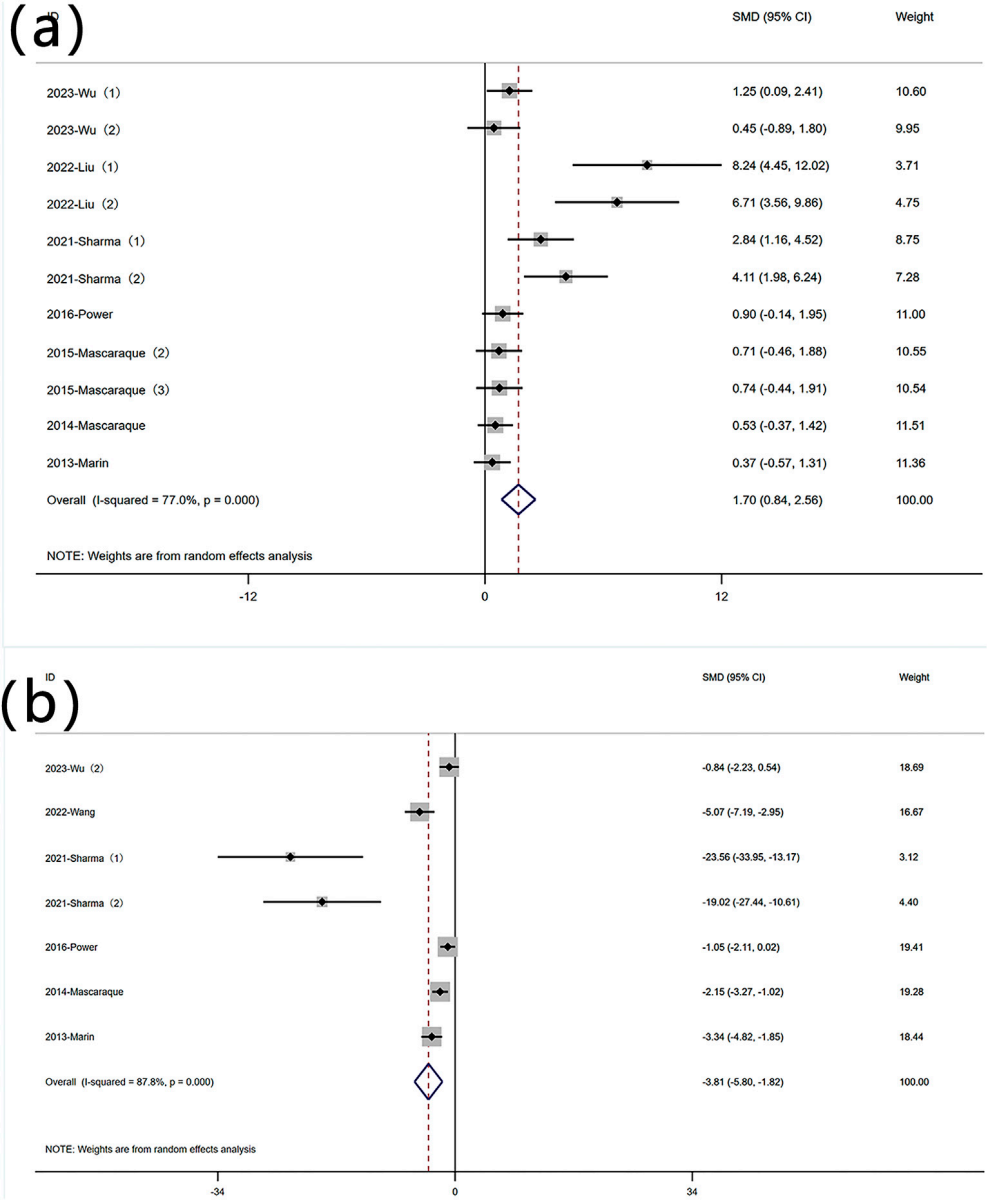
Forest plot of the effect of rutin on IBD, outcome measure: **(a)** weight loss. **(b)** disease activity index.

The reduction in body weight is a core symptom of IBD. A total of 11 datasets were used to assess this indicator. A meta-analysis of body weight changes revealed significant heterogeneity (I^2^ = 77.0%, P < 0.05), and a random-effects model was used to conduct meta-analysis on weight change. Analysis of these studies showed that rutin has an effect on weight reduction [SMD: 1.70, P < 0.05; 95% CI(0.84,2.56)] (Figure 3 Forest plot of the effect of rutin on IBD, outcome measure: (a) weight loss. (b) disease activity index).

#### 3.4.2 DAI

DAI is an indicator used to measure the activity level of a disease by quantifying its clinical manifestations. Six experiments reported data on DAI. Significant heterogeneity was observed (I^2^ = 87.8%, P < 0.05), and a random-effects model was used for meta-analysis of DAI. Rutin can reduce DAI in mice induced with colitis [SMD: −3.81 P < 0.05; 95% CI(-5.80,-1.82)] (Figure 3 Forest plot of the effect of rutin on IBD, outcome measure: (a) weight loss. (b) disease activity index).

#### 3.4.3 Oxidative stress indicators

##### 3.4.3.1 MPO content

**FIGURE 4:**
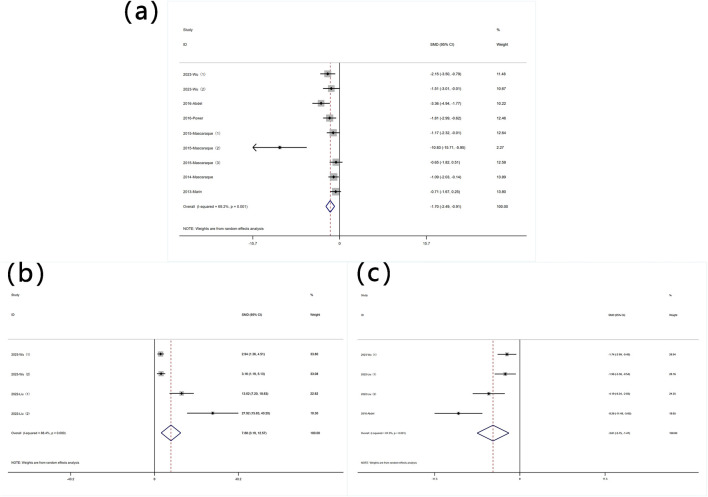
Forest plot of the effect of rutin on IBD, outcome measure: **(a)** MPO **(b)** SOD **(c)** MDA.

Nine experiments quantified MPO activity via protein concentration measurements. Significant heterogeneity was observed (I^2^ = 69.2%, P < 0.05), and a random-effects model was used for meta-analysis of weight change. Rutin can reduce MPO in mice induced with colitis [SMD: −1.70, P < 0.05; 95% CI (−2.49, −0.91)] (Figure 4 Forest plot of the effect of rutin on IBD, outcome measure: (a) MPO (b) SOD (c) MDA.

##### 3.4.3.2 SOD content

Four experiments reported SOD levels. Significant heterogeneity was observed (I^2^ = 88.4%, P < 0.05), and a random-effects model was used for meta-analysis of SOD. Rutin can increase SOD levels in experimental animals induced with colitis [SMD: 7.88, P < 0.05; 95% CI (3.19, 12.57)] (Figure 4 Forest plot of the effect of rutin on IBD, outcome measure: (a) MPO (b) SOD (c) MDA.

##### 3.4.3.3 MDA content

Four experiments reported MDA levels. Significant heterogeneity was observed (I^2^ = 81.9%, P < 0.05), and a random-effects model was used for meta-analysis of MDA. Rutin can decrease MDA levels in experimental animals induced with colitis [SMD: −3.61, P < 0.05; 95% CI (−5.75, −1.47)] (Figure 4 Forest plot of the effect of rutin on IBD, outcome measure: (a) MPO (b) SOD (c) MDA.

#### 3.4.4 Inflammatory indicators

##### 3.4.4.1 TNF-α content

**FIGURE 5:**
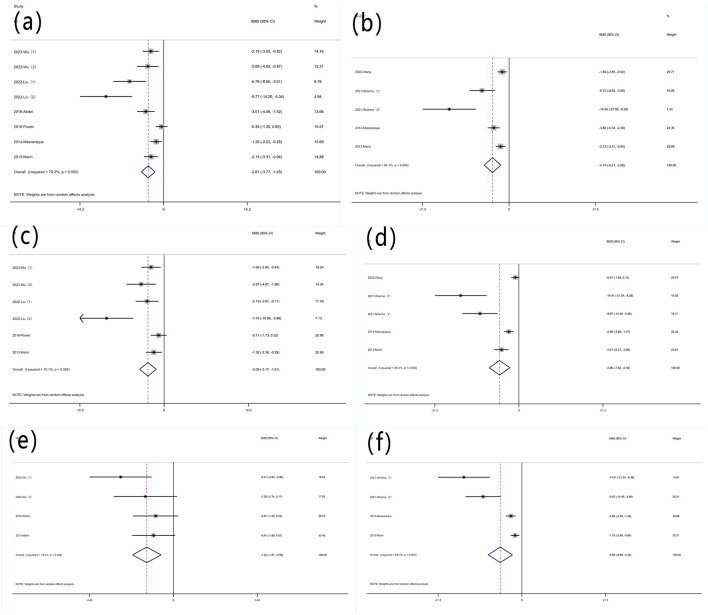
Forest plot of the effect of rutin on IBD, outcome measure: **(a)** TNF-α **(b)** TNF-α mRNA **(c)** IL-6 **(d)** IL-6 mRNA **(e)** IL-1β **(f)** IL-1β mRNA.

Nine experiments reported protein levels of TNF-α. Significant heterogeneity was observed (I^2^ = 79.2%, P < 0.05), and a random-effects model was used for meta-analysis of TNF-α[SMD: −2.61, P < 0.05; 95% CI (−3.77, −1.45)]. Five experiments quantified TNF-α mRNA levels (I^2^ = 84.1%, P < 0.05) [SMD: −4.14, P < 0.05; 95% CI (−6.21, −2.06)]. Rutin can decrease TNF-α levels in experimental animals induced with colitis (Figure 5A).

##### 3.4.4.2 IL-6 content

Six experiments reported protein levels of IL-6. Heterogeneity was significant (I^2^ = 70.1%, P < 0.05), and a random-effects model was used for meta-analysis of IL-6 [SMD: −2.09, P < 0.05; 95% CI (−3.17, 1.01)]. five experiments quantified IL-6 mRNA levels, (I^2^ = 89.4%, P < 0.05) [SMD: −4.96, P < 0.05; 95% CI (−7.62, 2.30)]. Rutin can decrease IL-6 levels in experimental animals induced with colitis (Figure 5B).

##### 3.4.4.3 IL-1β content

Four experiments reported IL-1β levels. Significant heterogeneity was not observed (I^2^ = 18.4%, P = 0.299), and a random-effects model was used for meta-analysis of IL-1β [SMD: −1.22, P = 0.299; 95% CI (−1.87, −0.56)]. Four additional experiments quantified IL-1β mRNA levels, (I^2^ = 88.4%, P < 0.05) [SMD: −5.56, P= < 0.05; 95% CI (−8.69, −2.42)]. Rutin can decrease IL-1β levels in experimental animals induced with colitis (Figure 5C).

#### 3.4.5 Intestinal permeability

**FIGURE 6:**
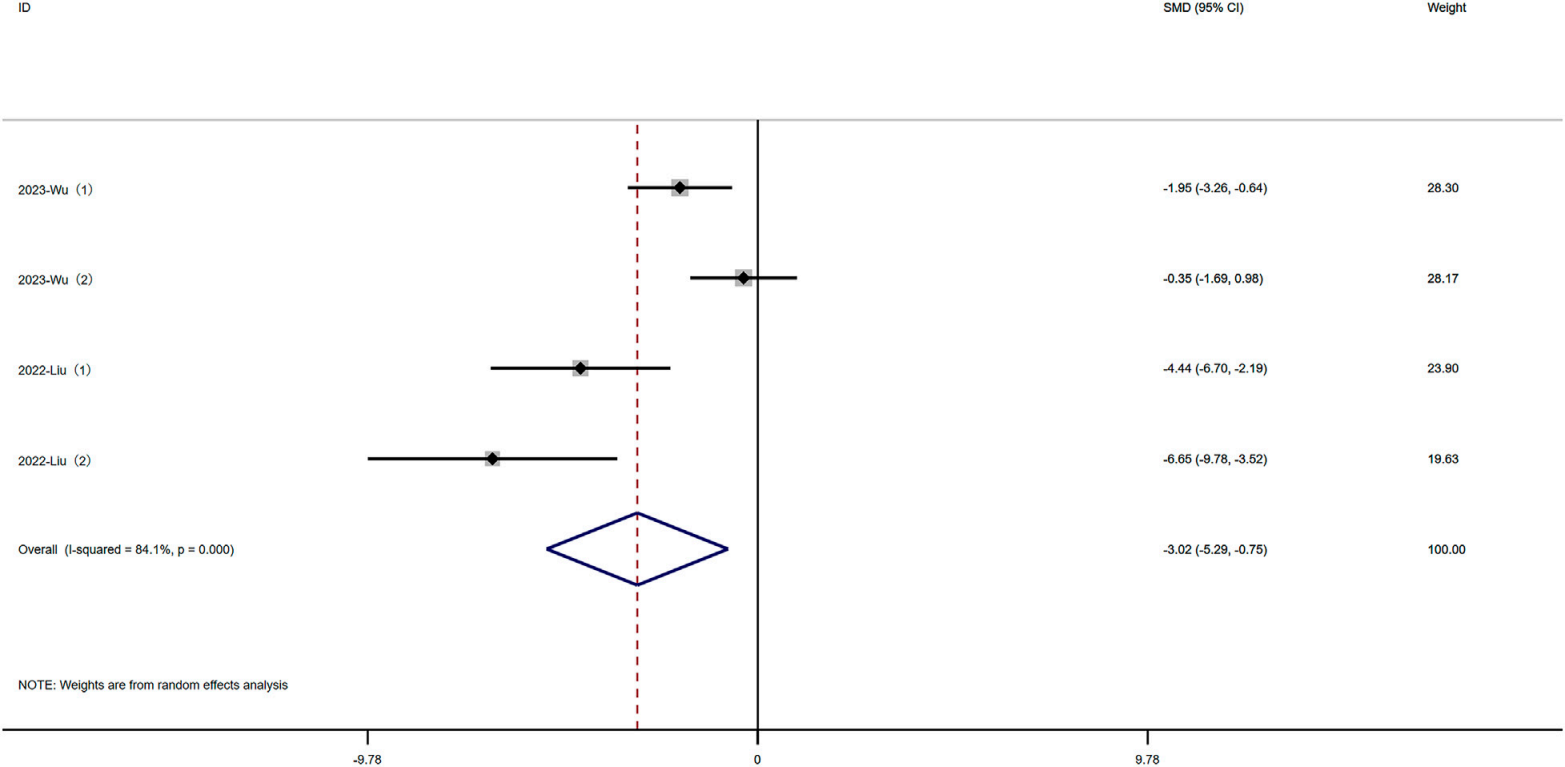
Forest plot of the effect of rutin on IBD, outcome measure: FITC.

Fluorescein isothiocyanate (FITC) is a fluorescein derivative used to detect intestinal permeability. Four experiments reported FITC levels. Significant heterogeneity was observed (I^2^ = 84.1%, P < 0.05), and a random-effects model was used for meta-analysis of FITC. Rutin can decrease FITC levels in experimental animals induced with colitis [SMD: −3.02, P < 0.05; 95% CI (−5.29, −0.75)](Figure 6 Forest plot of the effect of rutin on IBD, outcome measure: FITC.

### 3.5 Subgroup analysis

We performed subgroup analysis on the primary outcome indicators as planned.

#### 3.5.1 Weight loss

**FIGURE 7:**
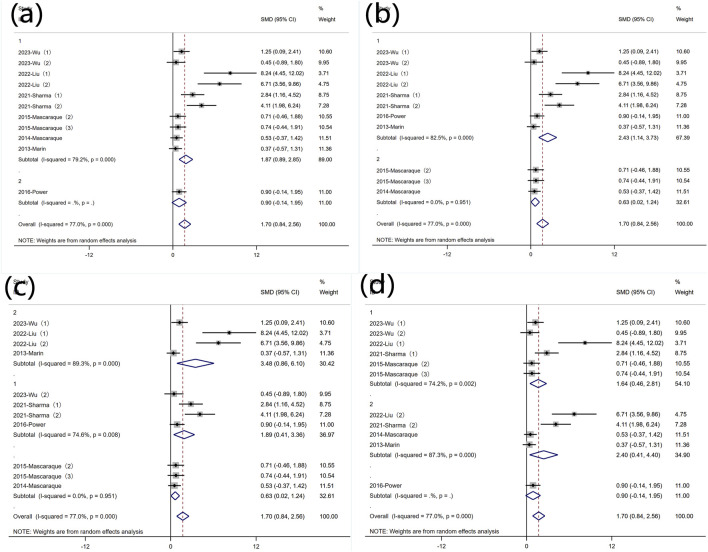
Subgroup analysis of the effect of rutin on weight loss: **(a)** animal gender **(b)** modeling method **(c)** modeling dosage **(d)** treatment dosage.

Due to significant heterogeneity, subgroup analyses were conducted based on predefined categories: animal gender, modeling method, modeling dosage, and treatment dosage. The results indicated that animal gender was not a primary source of heterogeneity (Figure 7 Subgroup analysis of the effect of rutin on weight loss: (a) animal gender (b) modeling method (c) modeling dosage (d) treatment dosage. In eight experiments using DSS-induced colitis, significant heterogeneity was observed. Three experiments utilized other modeling methods, exhibiting non-significant heterogeneity and non-statistically significant conclusions (Figure 7 Subgroup analysis of the effect of rutin on weight loss: (a) animal gender (b) modeling method (c) modeling dosage (d) treatment dosage. Excluding three experiments that did not involve DSS, subgroup analysis of the modeling dosages in the remaining eight experiments still revealed high heterogeneity (Figure 7 Subgroup analysis of the effect of rutin on weight loss: (a) animal gender (b) modeling method (c) modeling dosage (d) treatment dosage. Subgroup analysis was conducted based on different rutin doses, except for one experiment without detailed dose data (Power et al., 2016), where six experiments had rutin doses below 40 mg/kg, and 4 experiments had doses above 40 mg/kg, showing significant heterogeneity (Figure 7 Subgroup analysis of the effect of rutin on weight loss: (a) animal gender (b) modeling method (c) modeling dosage (d) treatment dosage., yet rutin reduced weight loss in both subgroups.

#### 3.5.2 DAI

**FIGURE 8:**
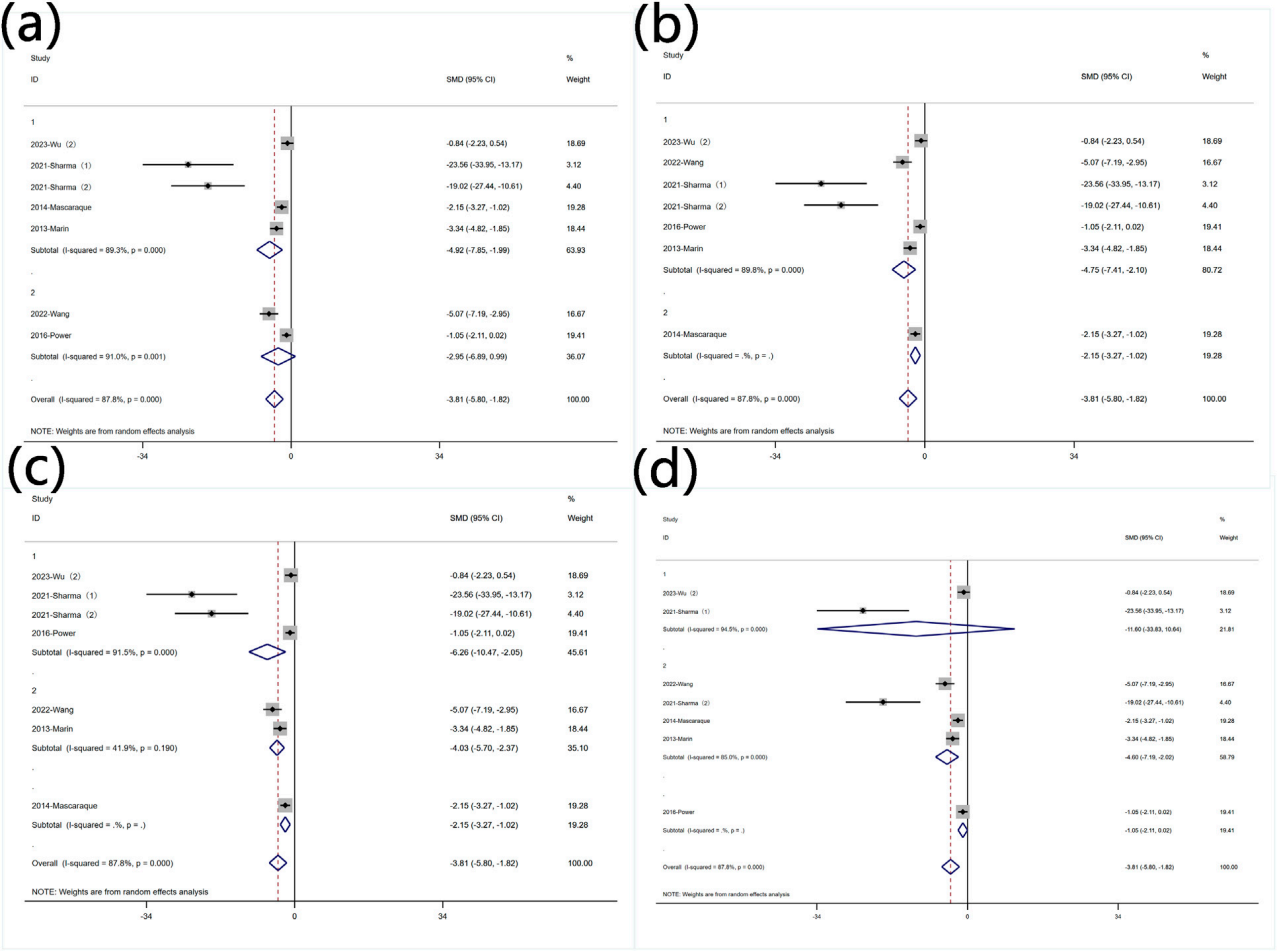
Subgroup analysis of the effect of rutin on DAI: **(a)** animal gender **(b)** modeling method **(c)** modeling dosage **(d)** treatment dosage.

Subgroup analysis was conducted based on predefined subgroups: according to different animal genders. Four experiments utilized female animals, while two used males. Both subgroups still exhibited high heterogeneity. Rutin significantly reduced DAI in the female subgroup, whereas the effect was not evident in the male subgroup (Figure 8 Subgroup analysis of the effect of rutin on DAI: (a) animal gender (b) modeling method (c) modeling dosage (d) treatment dosage. Six studies employed DSS for disease modeling, while one study utilized an alternative approach. Comparative analysis with the overall SMD demonstrated persistent significant heterogeneity (Figure 8 Subgroup analysis of the effect of rutin on DAI: (a) animal gender (b) modeling method (c) modeling dosage (d) treatment dosage. Regarding the different modeling doses, four experiments utilized DSS doses less than 2.5% (wt/v), showing significant heterogeneity, while two experiments used doses greater than 2.5% (wt/v) with insignificant heterogeneity. Rutin reduced DAI values in both subgroups (Figure 8 Subgroup analysis of the effect of rutin on DAI: (a) animal gender (b) modeling method (c) modeling dosage (d) treatment dosage. Subgroup analysis based on different doses of rutin showed that, except for one experiment that did not specify the dose, two experiments used rutin doses less than 40 mg/kg, while four experiments used doses greater than 40 mg/kg. Both subgroups exhibited significant heterogeneity. Rutin significantly reduced DAI values in the high-dose subgroup, while the effect was not evident in the low-dose subgroup (Figure 8 Subgroup analysis of the effect of rutin on DAI: (a) animal gender (b) modeling method (c) modeling dosage (d) treatment dosage.

#### 3.5.3 Protein levels of MPO

**FIGURE 9:**
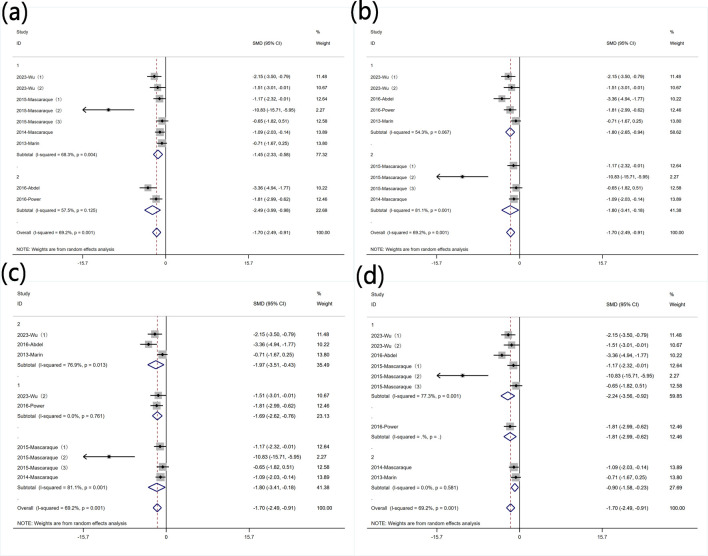
Subgroup analysis of the effect of rutin on MPO: **(a)** animal gender **(b)** modeling method **(c)** modeling dosage **(d)** treatment dosage.

Subgroup analysis was conducted based on predefined subgroups: according to different animal genders. Seven experiments utilized female animals, while two used males. Both subgroups still exhibited high heterogeneity. Rutin reduced MPO levels in both subgroups (Figure 9 Subgroup analysis of the effect of rutin on MPO: (a) animal gender (b) modeling method (c) modeling dosage (d) treatment dosage. Subgroup analysis was conducted based on different modeling methods. Both subgroups still exhibited high heterogeneity. Rutin was more effective in reducing MPO levels in the subgroup that used DSS for modeling (Figure 9 Subgroup analysis of the effect of rutin on MPO: (a) animal gender (b) modeling method (c) modeling dosage (d) treatment dosage. Subgroup analysis was conducted based on different modeling doses. Except for four experiments that did not utilize DSS for modeling, two experiments used DSS doses less than 2.5% (wt/v) with insignificant heterogeneity, while three experiments used doses greater than 2.5% (wt/v) with significant heterogeneity. Rutin reduced MPO levels in each subgroup (Figure 9 Subgroup analysis of the effect of rutin on MPO: (a) animal gender (b) modeling method (c) modeling dosage (d) treatment dosage. Subgroup analysis was conducted based on different rutin doses. Except for one experiment that did not specify the rutin dose, six experiments used rutin doses less than 40 mg/kg, and two experiments used doses greater than 40 mg/kg. Significant heterogeneity was observed exclusively in the low-dose rutin subgroup. Rutin reduced MPO levels in both groups (Figure 9 Subgroup analysis of the effect of rutin on MPO: (a) animal gender (b) modeling method (c) modeling dosage (d) treatment dosage.

#### 3.5.4 Protein levels of TNF-α

Subgroup analysis was conducted based on predefined subgroups

**FIGURE 10:**
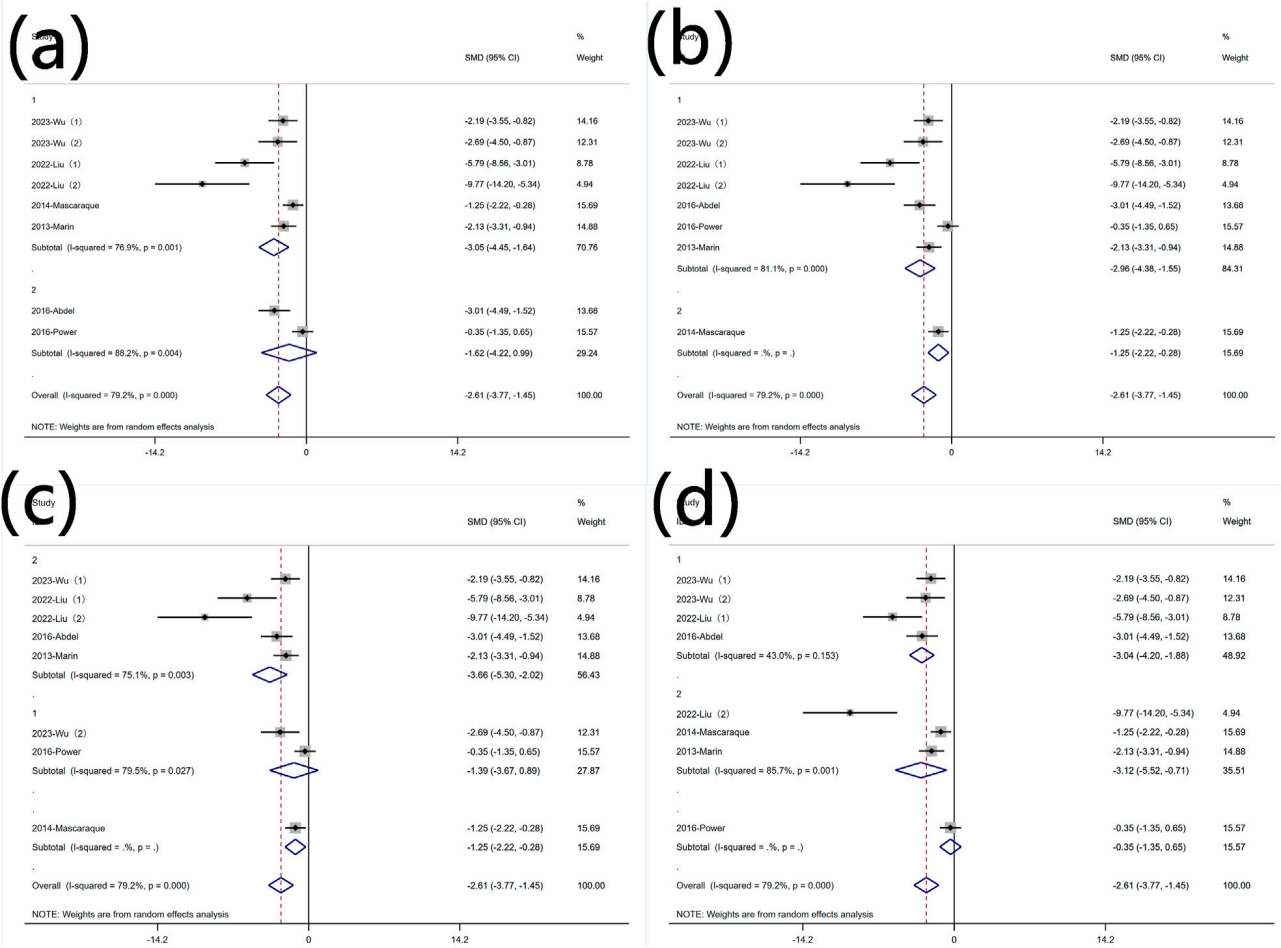
Subgroup analysis of the effect of rutin on TNF-α: **(a)** animal gender **(b)** modeling method **(c)** modeling dosage **(d)** treatment dosage.

According to different animal genders. Six experiments utilized female animals, while three used males. Both subgroups still exhibited high heterogeneity (Figure 10 Subgroup analysis of the effect of rutin on TNF-α: (a) animal gender (b) modeling method (c) modeling dosage (d) treatment dosage. Rutin reduced TNF-α levels in both subgroups. Subgroup analysis was conducted based on different modeling methods. Only one experiment did not use DSS for modeling, and in the experiments with DSS for modeling, heterogeneity remained high (Figure 10 Subgroup analysis of the effect of rutin on TNF-α: (a) animal gender (b) modeling method (c) modeling dosage (d) treatment dosage. Subgroup analysis was conducted based on different modeling doses. Except for one experiments that did not utilize DSS for modeling, two experiments used DSS doses less than 2.5% (wt/v), five experiments used doses greater than 2.5% (wt/v) with significant heterogeneity (Figure 10 Subgroup analysis of the effect of rutin on TNF-α: (a) animal gender (b) modeling method (c) modeling dosage (d) treatment dosage. Rutin can reduce the content of TNF-α in both subgroups, but only the group with the high dosage for model establishment shows significant significance. Subgroup analysis was conducted based on different rutin doses. Except for one experiment that did not specify the rutin dose, four experiments used rutin doses less than 40 mg/kg did not show significant heterogeneity, and four experiments used doses greater than 40 mg/kg exhibited significant heterogeneity. Rutin reduced TNF-α levels in both groups (Figure 10 Subgroup analysis of the effect of rutin on TNF-α: (a) animal gender (b) modeling method (c) modeling dosage (d) treatment dosage.

### 3.6 Publication bias

Conduct a publication bias analysis for the primary outcome measures. The funnel plot indicates the presence of publication bias. Egger’s regression tests were conducted to assess publication bias for each outcome measure, revealing significant publication bias for most primary outcome measures. However, after adjustment through the trim-and-fill method, the conclusions remained unchanged. (The relevant charts are in the Supporting Information).

### 3.7 Sensitivity analysis

Sensitivity analysis was conducted on at least 6 outcome measures included in the research reports, integrating the remaining studies by systematically excluding each study. The results of the analysis remained consistent after the systematic exclusion of each trial. (The relevant charts are in the Supporting Information).

## 4 Discussion

In our meta-analysis, the research results demonstrate that rutin has a significant effect on the treatment of experimental animals induced with IBD. Rutin can reduce weight loss, DAI scores, inflammatory marker MPO (Myeloperoxidase), TNF-α, IL-1β, IL-6, mRNA-INOS, MDA, FITC, while also increasing SOD. This suggests that rutin can reduce intestinal damage in experimental animals with IBD, decrease inflammation, and mitigate oxidative stress. Subgroup analysis was conducted on the primary outcome measures, and the results indicate that the modeling method and dosage may be sources of heterogeneity for each outcome measure. Sensitivity analysis of the primary outcome measures demonstrates good stability of the results.

5-Aminosalicylic acid (5-ASA) has been demonstrated to inhibit NF-κB activity and scavenge reactive oxygen species, making it a first-line treatment for inflammatory bowel disease (IBD). However, considering its limitations, including drug side effects, resistance, and variable efficacy, there is a need for alternative therapies (Liu et al., 2023). Herbal extracts of natural origin are generally considered safe and well-tolerated. Given that the pathogenesis of IBD results from the interaction between dietary factors and behavioral risk factors (Crooks et al., 2022), changes in lifestyle and dietary patterns will play a crucial role in preventing and ameliorating IBD. We believe this finding has significant public health implications.

### 4.1 Potential Mechanisms of Rutin

**FIGURE 11:**
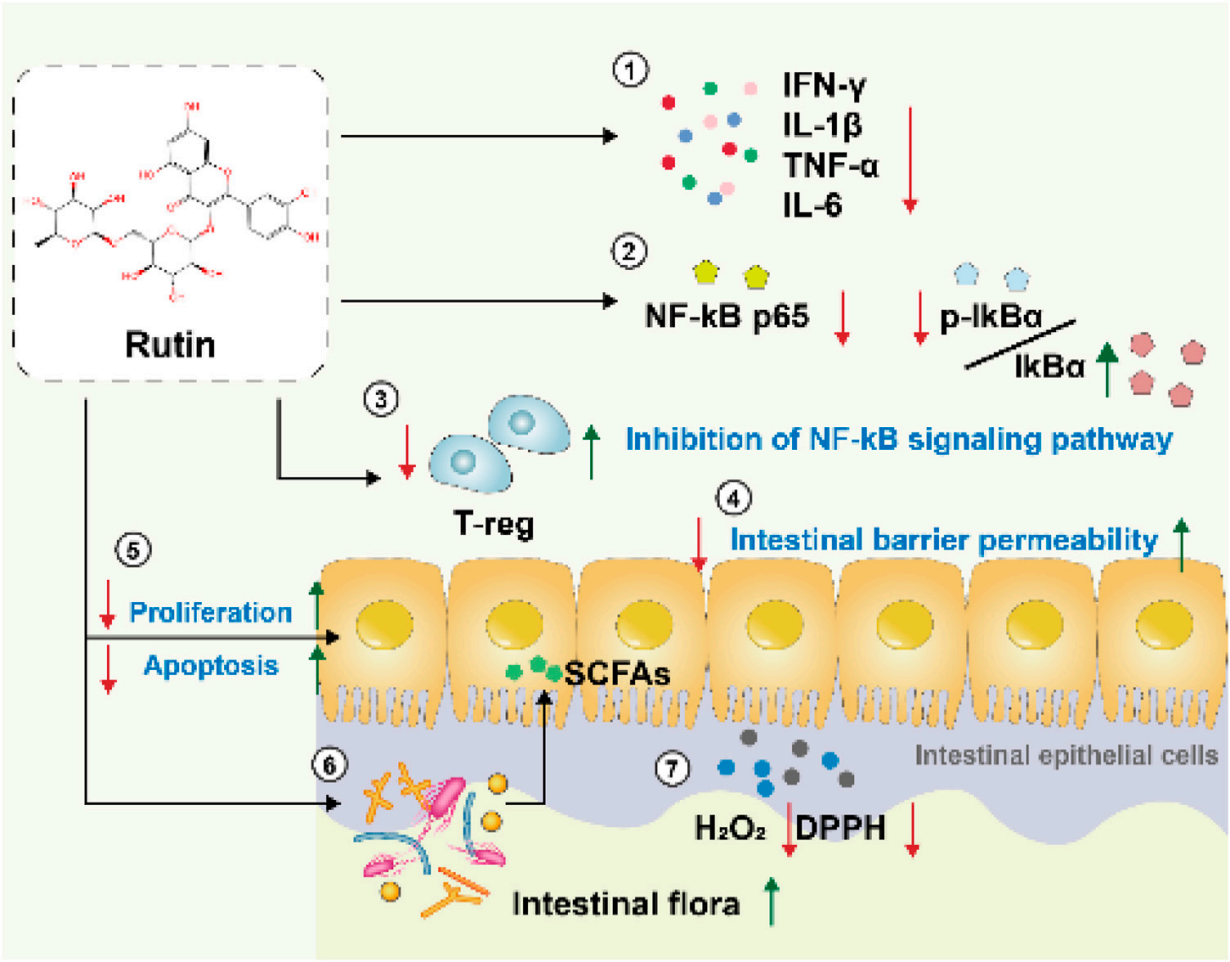
Potential mechanisms of rutin.

We summarizes potential mechanisms by which rutin may exert effects on inflammatory bowel disease (IBD), based on an analysis of the included literature (Figure 11):

#### 4.1.1 Anti-inflammatory effects

Through real-time quantitative PCR analysis, rutin significantly reduces the expression levels of crucial inflammatory cytokines, including interferon-gamma (IFNγ), interleukin-1 beta (IL-1β), interleukin-6 (IL-6), and tumor necrosis factor-alpha (TNF-α), decreasing the production of pro-inflammatory factors by over 80%. These factors play a critical role in inflammatory bowel diseases, and their reduction contributes to alleviating intestinal inflammation (Mascaraque et al., 2014; Wu et al., 2023). Moreover, rutin effectively suppresses the production of nitric oxide (NO) and prostaglandin E2 (PGE2) in colonic tissues, as well as decrease the expression of inducible nitric oxide synthase (iNOS) and cyclooxygenase-2 (COX-2). Rutin also significantly decreases the activity of MPO in dss-induced acute colitis mice (Marín et al., 2013). At the biochemical level, the rutin metabolites 3,4-dihydroxyphenylacetic acid (DHPAA) and 3,4-dihydroxytoluene (DHT) possess vicinal hydroxyl groups. These hydroxyl groups undergo condensation reactions with carbonyl groups in reactive carbonyl species (RCS), such as glyoxal and methylglyoxal, forming stable adducts. This reaction depletes reactive carbonyl species (RCS), thereby limiting their capacity to participate in glycation reactions with amino acid residues in proteins. Consequently, it inhibits the formation of advanced glycation end-products (AGEs) and suppresses inflammation (Pashikanti et al., 2010).

### 4.2 Antioxidant effects

In patients with IBD, there is a significant increase in H2O2 levels in the colonic mucosa. The ability to remove H2O2 has certain implications for the treatment of IBD. Meanwhile, DPPH is a highly stable free radical that can induce oxidative stress, leading to damage to biomolecules in the body. Within 30 min, RT achieves a clearance rate of 70% for both H2O2 and DPPH, and the clearance rate is dose-dependent (Wu et al., 2023). This demonstrates that rutin can alleviate damage caused by IBD through its antioxidant properties.

#### 4.2.1 Inhibition of the NF-κB signaling pathway

The NF-κB signaling pathway is a crucial mediator in the development and progression of inflammatory bowel disease (IBD). This pathway plays a pivotal role in regulating the immune response to infection and inflammation. In response to inflammatory stimuli, NF-κB is activated by phosphorylation of its inhibitor, IκB, leading to IκB degradation and allowing NF-κB to translocate to the nucleus. Within the nucleus, NF-κB binds to specific DNA sequences, triggering the transcription and translation of various pro-inflammatory cytokines and mediators, such as TNF-α, IL-1β, and IL-6, which play crucial roles in the inflammatory processes of IBD (Liu et al., 2022). Through Western blot analysis, the study found that rutin can directly inhibit the binding of p65 to DNA (Kim et al., 2005) and alter the cytoplasmic ratio of p-IκBα and IκBα. Additionally, rutin significantly inhibits the activation of MAPKs-NF-κB, PI3K-Akt, MK2, and GSK3ß. It prevents various oxidative inflammatory signaling cascades and kinases, such as p38/MK2 and PI3K/Akt/GSK3β/MAPKs, from activating the classical inflammatory transcription factor NF-κB to exacerbate the inflammatory response. This suggests that rutin may alleviate DSS-induced colitis by influencing the NF-κB signaling pathway (Sharma et al., 2021; Liu et al., 2022).

#### 4.2.2 Restoring the dysbiosis of gut microbiota in colitis mice

The gut microbiota plays a fundamental role in maintaining intestinal health and is crucial in the pathophysiology of inflammatory bowel disease (IBD). One of the primary functions of the gut microbiota is to support the integrity of the epithelial barrier. When the epithelial barrier is compromised, endotoxins (such as lipopolysaccharides) are more likely to translocate into colonic tissues. Once endotoxins enter the colonic tissues, they activate immune cells such as Kupffer cells (liver macrophages) and neutrophils. This activation stimulates the NF-κB signaling pathway, leading to a significant increase in the production of pro-inflammatory cytokines, including tumor necrosis factor-alpha (TNF-α) and interleukin-6 (IL-6) (Liu et al., 2022).

Some studies have found through high-throughput sequencing of 16S rRNA that rutin can restore the decreased abundance of gut microbiota caused by IBD (such as Faecalibaculum rodentium and Turicibacter) and reduce the abundance of potentially disease-associated bacteria (such as Romboutsia and the Escherichia-Shigella group). The enrichment of Escherichia-Shigella is a potential characteristic of IBD patients and an initiator of IBD pathogenesis, including the stimulation of mucin degradation by proteases, adhesion and invasion of intestinal epithelial cells, and promotion of the secretion of pro-inflammatory cytokines and chemokines (Liu et al., 2022) (Mazzarella et al., 2017). Additionally, rutin can be metabolized by gut microbiota into microbial-derived metabolites with anti-inflammatory, antioxidant, and colonic barrier-protective properties, which may contribute to the production of SCFAs in colitis mice (Power et al., 2016; Liu et al., 2022).

#### 4.2.3 Restore epithelial integrity and reduce tissue damage

Rutin can regulate tight junction proteins, mucin secretion proteins, epithelial cell proliferation, and apoptosis, which contribute to maintaining the integrity of the intestinal barrier (Sharma et al., 2021). During the recovery phase of colitis, rutin significantly enhances the restoration of colonic crypts and goblet cells while reducing the activity of colonic myeloperoxidase (MPO). (Power et al., 2016).

#### 4.2.4 Inhibit adaptive immune responses

Rutin has immunomodulatory potential and can suppress the exacerbation of inflammation in adaptive immune responses by influencing Treg cells in the spleen, thus alleviating inflammation (Sharma et al., 2021).

#### 4.2.5 Restore intestinal permeability

High intestinal permeability is a primary characteristic of leaky gut syndrome, which allows food antigens, symbiotic or pathogenic bacteria, and bacterial components to enter the lamina propria and subsequently the bloodstream, triggering systemic inflammation described in various disease conditions (Vanuytsel et al., 2021). In scientific experiments, intestinal permeability is typically assessed using fluorescent dextrans such as fluorescein isothiocyanate (FITC)-labeled dextran and tetramethylrhodamine B isothiocyanate (TRITC)-labeled dextran (Woting and Blaut, 2018). The two included articles demonstrated that rutin can improve intestinal permeability in mouse models of IBD (Liu et al., 2022; Wu et al., 2023).

### 4.3 Rutin’s extended effects

#### 4.3.1 Comparison of rutin with commonly used pharmaceutical medicines

Among the included studies, dexamethasone (DEX) markedly reduced reactive oxygen species (ROS) levels to 1.1-fold, whereas rutin exhibited a more modest reduction (2.8-fold) (Wu et al., 2023). In contrast, comparative analyses between sulfasalazine (SASP) and rutin revealed comparable efficacy in mitigating weight loss, lowering disease activity index (DAI), and controlling inflammatory cytokines (e.g., TNF-α, IL-6) (Marín et al., 2013; Liu et al., 2022). Findings indicated that rutin achieved effects equivalent to 5-aminosalicylic acid (5-ASA) in improving DAI, colon length, and histopathological damage, with superior performance in specific metrics such as suppressing immunoglobulin levels (Sharma et al., 2021). Furthermore, rutin demonstrated anti-inflammatory potency comparable to budesonide in reducing pro-inflammatory cytokine secretion, while outperforming budesonide in attenuating weight loss and mortality rates (Mascaraque et al., 2014; Mascaraque et al., 2015).

#### 4.3.2 The therapeutic effects of rutin on other diseases

Studies have demonstrated that rutin alleviates streptozotocin (STZ)-induced inflammation and mitigates sporadic Alzheimer’s-type dementia by modulating pro-inflammatory mediators such as cyclooxygenase-2 (COX-2) and interleukin-8 (IL-8) (Javed et al., 2012; Sun et al., 2021)

Additionally, rutin exerts therapeutic benefits in diabetes management by reducing intestinal carbohydrate absorption, stimulating insulin secretion from pancreatic β-cells, preventing degeneration of Langerhans islets, enhancing tissue glucose uptake, and suppressing hepatic gluconeogenesis (Ghorbani, 2017). These mechanisms collectively contribute to its efficacy in treating diabetes and associated complications. Furthermore, emerging evidence suggests rutin’s potential in addressing obesity and acute lung injury through its anti-inflammatory and metabolic regulatory properties (Yuan et al., 2017; Yeh et al., 2014).

### 4.4 Strengths and limitations of this meta-analysis

This study is the first article to establish a connection between rutin and inflammatory bowel disease (IBD), and to conduct a systematic review and meta-analysis of relevant animal experiments. To provide a comprehensive analysis, all eligible studies were included from the inception of the databases up to the present. The primary goal was to synthesize existing preclinical evidence to better understand rutin’s potential as a therapeutic agent for IBD. However, there are some limitations to the current meta-analysis. One notable limitation of this study is the restriction to English-language databases and high-quality literature. This focus might have led to the exclusion of relevant studies published in other languages, potentially introducing a language bias and limiting the comprehensiveness of the review. Secondly, the overall quality of the included studies was found to be low. Although almost all studies mentioned “random” grouping of animals, they often lacked detailed descriptions of the specific randomization methods used. The analysis also indicated the presence of publication bias within the included literature. This bias suggests that studies with positive results might be more likely to be published. Consequently, the results should be interpreted with caution, taking into account the potential influence of this bias.

## 5 Conclusion

The current meta-analysis demonstrates that rutin can reduce weight loss, DAI, MPO, TNF-α, MDA, iNOS, IL-6, IL-1β, and FITC, while promoting SOD. The potential mechanisms underlying these protective effects include anti-inflammatory, antioxidant, inhibition of inflammatory signaling pathways, barrier protection, suppression of adaptive immune responses, restoration of intestinal permeability, and modulation of gut microbiota. Rutin has been substantiated by multiple lines of evidence as an ideal therapeutic agent for IBD. However, the overall methodological quality of the included studies is relatively low, and the analysis included a limited number of studies, which affects the generalizability of the results. Discrepancies may exist between the results observed in controlled experimental settings and actual situations. The promising results from animal studies need to be validated through rigorous, comprehensive, and methodologically sound research, including well-designed clinical trials.

## Data Availability

The raw data supporting the conclusion of this article will be made available by the authors, without undue reservation.
